# The Perceived Influence of Neurofibromatosis Type 1(NF1) on the Parents’ Relationship

**DOI:** 10.3390/children10030448

**Published:** 2023-02-25

**Authors:** Lori Wiener, Sima Zadeh Bedoya, Archita Goyal, Mallorie Gordon, Natalie Deuitch, Brigitte Widemann

**Affiliations:** 1 Pediatric Oncology Branch, Center for Cancer Research, National Institutes of Health, Bethesda, MD 20892, USA; 2 Tufts University School of Medicine, Boston, MA 02111, USA; 3 Translational and Functional Genomics Branch, National Human Genome Research, National Institutes of Health, Bethesda, MD 20892, USA

**Keywords:** Neurofibromatosis type I (NF1), parents, couples, dyadic coping, marriage, partnership, counseling

## Abstract

Neurofibromatosis type 1 (NF1) is a genetic condition affecting 1 in 3000 individuals. Having a child with a chronic illness can introduce both practical and emotional challenges to a parental relationship. This cross-sectional study was administered to 50 parents of children with NF1, diagnosed between the ages of 1–24. Each participant was provided a 50-item self-report survey to complete during an inpatient or outpatient visit. The survey gathered information on the participants’ views of the spouse/partner relationship, coping mechanisms, and elements that supported emotional connections. While the majority of parental relationships were reported to remain strong, the mean relationship quality was perceived to have decreased compared to prior to the child’s diagnosis. Compassionate and open communication, shared perspective, having time alone with their partner outside of medical situations, and dyadic coping were identified as strategies that could strengthen the relationship. The identified stressors to the parental relationship during the NF1 illness trajectory can inform interventions and help guide development of a couple’s intervention. The National Cancer Institute, NIH Institutional Review Board approved this study (12-C-0206).

## 1. Introduction

Neurofibromatosis type 1 (NF1) is a genetic condition affecting 1 in 3000 individuals [1,2]. Approximately 50% of cases are inherited from a parent while the other half are de novo [1]. NF1 is characterized by clinical features in almost all patients, most notably, the development of disfiguring, cutaneous neurofibromas, café-au-lait spots, macules, plexiform neurofibromas, and boney manifestations such as tibial dysplasia and scoliosis. [3]. NF1 causes tumors to grow along the nerves and is associated with an increased risk for the development of other cancers compared to the general population. In addition, NF1 often contributes to social and behavioral problems, including learning difficulties, development of Attention Deficit Hyperactivity Disorder (ADHD), and impaired social skills [4,5]. The manifestations of NF1 vary widely, even within families, from very mild to severely debilitating and manifestations often progress over time.

The presence of NF1 can have a significant impact on a child’s psychological health and quality of life [5,6,7,8]. Faced with concerns regarding their child’s appearance, tumors, social and behavioral issues, and uncertainty about the future [3,8], NF1 can be a significant source of stress for parents. These issues, along with trying to manage daily life, can also put considerable strain on couples [4]. While data support a strong, positive association between family functioning and a child’s psychological health [9,10] and a negative impact on a child’s mental health when inter-partner conflicts exist [11,12], we found no studies that explored the impact of an NF1 diagnosis on the parental relationship.

Several studies have been conducted among parents who have a child with a chronic illness including spina bifida, diabetes, and more acute conditions such as cancer [13,14,15]. A literature review was performed exploring the impact of a pediatric cancer diagnosis on couple functioning found overall strong adaptation in the areas of emotional closeness and general marital adjustment though difficulties in the domain of sexual intimacy and reports on conflict [16,17]. Studies have also demonstrated that dyadic coping strategies have helped couples cope better. Dyadic coping refers to the extent to which parents deal with stress as a dyad and has been identified as having a key role in individual and relationship functioning within couples facing significant stressors [18,19].

While there are many studies that examine the impact of childhood chronic illness on the family, factors that foster or inhibit parental relationship outcomes have received considerably less research attention. This cross-sectional study was designed to explore the impact of a child’s NF1 on the parents’ relationship by examining various aspects of the relationship before and after the NF1 diagnosis was made. We also aimed to explore dyadic adjustment, participant-identified issues of greatest stress individually and in the relationship, and interest in and challenges associated with couples counseling.

## 2. Materials and Methods

### 2.1. Participants

Eligibility for the study required that at least three months had elapsed since the child’s NF1 diagnosis and that the parent had been in a partnered relationship at time of diagnosis. The diagnosis of NF1 was based on established clinical criteria. Confirmation of diagnosis using genetic germline testing was performed when a diagnosis could not be clearly made based on the clinical diagnostic criteria. Eligibility was extended to both parents, regardless of their relationship status at the time of study enrollment.

### 2.2. Procedures

Study enrollment occurred when the child was present at the institution for scheduled appointments. Sixty-eight parents were approached. Four did not meet eligibility criteria. Of the eligible participants, 10 consented but did not complete the survey, 1 was not interested, 2 felt unable to participate due to lack of partner participation in child’s life or felt too much time had passed since their child was first diagnosed, and 1 had discrepant responses that could not be resolved. This resulted in a total cohort of 50 participants, and a 78% response rate. Each participant was provided a self-report survey to complete during an inpatient or outpatient visit after reviewing and signing the informed consent. The study was approved by the Institutional Review Board at the National Cancer Institute, National Institutes of Health.

### 2.3. Measures

The study team developed a 50-item self-report survey, including both multiple choice and open-ended questions for the purposes of this study. The survey collected information on demographics, as well as the participant’s view of their partner relationship, coping mechanisms, and stressors experienced secondary to the child’s NF1 (see Supplementary Material).

To assess marital/relationship issues, the survey included The Revised Dyadic Adjustment Scale (RDAS), a standard measure of marital stress that gauges aspects of marital adjustment that are tested including consensus (e.g., decision making), cohesion (e.g., working together and discussion), and marriage satisfaction (e.g., frequency of quarrels and considering separation) [20], and is a revision of the 32-item Dyadic Adjustment Scale (DAS) [21]. When compared with the DAS, this measure has good construct validity. In addition, the RDAS has good internal consistency (Cronbach α = 0.90) and excellent split-half reliability (Guttman split half = 0.94 and Spearman–Brown split half = 0.95) with estimates larger than those of the DAS.

To gather information on perceived changes in the relationship, three items related to emotional connection and managing stress effectively as a couple were adapted from the Gottman-17. This is a clinical marriage screening tool [20] that has been widely validated and well utilized as a measure of marital stability and divorce prediction [22].

Clinical consensus alongside a literature review guided question development for constructs related to specific challenges throughout the illness trajectory where no appropriate validated measures existed. The survey was first administered to parents of children with cancer and later adapted to parents of children with NF1. As noted in a prior publication [13], survey questions were pilot tested with 5 parents of children undergoing cancer treatment. Investigators assessed respondent comprehension of each item, ease of recall of past experiences, and ability to report personal experience.

### 2.4. Analysis

Descriptive analyses allowed for sample characterization and definition of key variables. SPSS 28 software was used to perform statistical analyses.

Qualitative analyses were conducted on free-text, open-ended responses to the question, “What recommendations would you make to a couple whose child was recently diagnosed in terms of trying to keep their relationship strong”. Independent analysis was performed by three investigators (L.W., A.G., S.B.) who read and coded text, and identifying emergent themes. Common themes across each question were then compiled, modified, and finalized based on team consensus. A coding dictionary was subsequently developed and applied to all responses. Discrepancies were reconciled through group review of the coded material until consensus was reached.

## 3. Results

### 3.1. Sample Characteristics

Fifty (50) parents of children aged 6 to 24 diagnosed with NF1 participated in this study. Participants included 32 individual parents and 9 “dyads” (9 groups of 2 people who identified being a parent of the same child) and ranged in age from 27 to 72 (M = 46.22, SD = ±8.85). Each participant was treated as an individual (enrollment, questions, and analyses). Table 1 includes additional information, such as marital status. The majority (78%) self-reported their race as Caucasian.

### 3.2. Relationship Quality

Forty-two percent of participants indicated strengthened relationships secondary to their child’s NF1, while 20.0% reported the relationship was challenged but still strong, and another 20.0% reported no change. Since time of diagnosis, few (4.0%) participants had considered separation, and 14.0% had separated.

Most participants (86.0%) rated their relationship in the year prior to their child’s NF1 diagnosis as good/very good/excellent and 14.0% rated it as poor/fair. Similarly, the majority of participants (68.0%) rated the quality of their current relationship as good/very good/excellent, and 32.0% rated it as poor/fair.

Nearly half (42.0%) of participants indicated on the aforementioned questions that their relationship had moved in a negative direction. Treated as a continuous scale, on which “poor” = 1 and “excellent” = 5, participants’ responses demonstrated a decrease in the mean relationship quality over the previous year (3.70 vs. 3.16, *t* = 3.5, *p* = 0.001, Cohen = 0.50, indicating small-to-medium effect size).

### 3.3. Emotional Connection

Participant perception of their relationship pre- and post-diagnosis of their child’s NF1 was assessed using a 5-point scale (never, sometimes, often, almost always, and always), which was then dichotomized to never/sometimes and often/almost always/always for analysis. Significant differences were found with participants reporting more loneliness (mean diff = −0.58, *p* < 0.01), less intimacy (mean diff = 0.72, *p* <0.001), less shared decision-making (mean diff = 0.54, *p* < 0.01), more anger (mean diff = −0.30, *p* <.05), and more tension/stress in the relationship (mean diff = −0.44, *p* < 0.05).

Participants were also asked how they were currently handling six different intrapersonal aspects of their relationship. Participants either responded “not a problem”, “sometimes a problem”, or “often a problem”, and this was dichotomized to “not a problem” and “sometimes”/”often a problem”. Feeling taken for granted, spending time together, and staying emotionally in touch were reported as sometimes/often a problem in the relationship (64%, 64%, and 60%, respectively).

### 3.4. Most Stressful Issues to Self, Partner, and Relationship

Participants were asked about potential stressors impacting their partners, their relationship, and/or themselves. Overwhelmingly, participants reported that fear of disease outcome was most stressful to themselves, while financial issues and (to a lesser degree) lack of intimacy, were most stressful to the relationship. Additionally, participants reported financial concerns and fear of disease outcome as issues they believed to be most stressful to their partners. See Table 2.

### 3.5. Handling Stress

Twenty-seven participants (54%) indicated they were managing stress related to caring for their child with NF1 “somewhat” effectively with their partner, 16% reported handling their stress completely effectively, and 30% reported they were challenged in this area. To further clarify the experienced stressors, participants were asked to rate certain areas that could be problematic for their relationship as “not a problem”, “sometimes a problem”, or “often a problem”. Areas rated to be problematic “sometimes” or “often” by over half of the participants included helping each other reduce daily stress (sometimes: 52%, often: 16%), talking about daily stresses together (sometimes: 46%, often: 22%), talking together about stress in a helpful manner (sometimes: 42%, often: 20%), listening with understanding about each other’s stresses and worries (sometimes: 42%, often: 18%), and partner taking job or other stresses out on the other (sometimes: 38%, often: 20%).

### 3.6. What Would Strengthen Your Marriage/Relationship

Optional open- and closed-ended items were included to gather perspective on what would strengthen a couple’s relationship following a child’s NF1 diagnosis. Of those who endorsed practical suggestions in the closed question, participants most frequently suggested having the partner more involved in household chores (30%). Those who endorsed “other” (28%) tended to note that having their partner more involved with finances would strengthen their relationship.

Responses to the open-ended question, “What recommendations would you make to a couple whose child was recently diagnosed in terms of trying to keep their relationship strong” reflected three major themes reflecting the value participants placed on the interpersonal aspects of their partner relationship. The themes included open and respectfulcommunication, shared perspective, having time alone with partner outside of medical situations, and dyadic coping strategies.

### 3.7. Open and Compassionate Communication

Participants believed that open communication between themselves and their partner could help them understand their partner better and make their voices heard. The need for open communication permeated the responses as the following quote illustrates: “Talk frequently about your emotions. You can’t understand each other’s actions without understanding the emotions underneath”.

### 3.8. Sharing the Same Outlook

Respondents reflected on the value of a shared outlook on current and future goals and methods of reaching their shared goals whether that was through further communication, education of their child’s illness, a supportive medical team, or uniting based on faith. Examples included, “Focus on your child—that helped us to maintain a strong relationship” and “Find good doctors so they can help you make good informative decisions”.

### 3.9. Having Time Outside of Illness

Participants wrote about the importance of maintaining a strong family dynamic and emphasized the importance of maintaining a relationship with their partner that did not solely revolve around their child’s NF1. Examples included, “Put [your] relationship as a very high priority”; “It’s hard not to make a child’s NF1 the whole focus of one’s own life as a parent”; and “Be thankful for every day. Try not to stray from normalcy outside of appointments. Find ways to strengthen family bonds”.

### 3.10. Dyadic Strategies

Dyadic adjustment was reflected by recognizing partners’ feelings and stresses, respecting their opinions, being supportive, and understanding how the other partner experiences stresses. Illustrative examples included: “We deal with the stresses differently. Try to respect each other’s process”; “I can remember standing in the hospital waiting room crying, with him just holding us. It meant a lot”; “Finding each other approachable and that the other cares. Feeling heard and understood”; and “When we just cried together in each other’s arms”.

Participants also expressed that their relationship was strengthened by a capacity to be flexible, understand and adapt new family roles. The following quote illustrates this sentiment. “My wife has to take care of the business and home while I am in charge of taking care of our son and communications with the doctors and everyone else involved in his treatments and care. In these times we are divided but working together as a team to accomplish what needs to be done”.

### 3.11. Interest, Timing, and Location of Counseling Services

Interest in couples counseling to address strategies to support/strengthen their relationship if it were offered was gauged. Most (58%) responded “yes”, 12% responded “no”, and 30% were “not sure”. When asked about appropriate timing for services to be offered, the majority of participants responded, “soon after diagnosis” (28%) or “any point after diagnosis” (28%), while others said, “first year after diagnosis” (16%), “only if we ask” (14%), or “other” (14%). Most participants preferred that this service be offered in their home community (50%) or in the hospital (26%). When participants were asked to list topics they felt to be most important to address in counseling, the most common responses included open communication, financial guidance, knowing what to expect, understanding their spouse’s stress, and being heard.

Several barriers were reported pertaining to participating in couples counseling. These included financial concerns (58%), limited time (50%), difficulty of both partners to be present at the same time (24%), and difficulty of talking about relationship while child is receiving treatment (18%). Others believed their partner would never do it (16%), believed they did not need it (14%), or were not interested (8%).

## 4. Discussion

This study explored the effect that a child’s NF1 diagnosis has on the parental relationship. Findings indicate that most couples were able to identify some effective means of coping with their situation, given that many reported that their relationship either stayed the same or improved over time. This differs from findings among parents of children with cancer, over half of whom reported that their relationship had been challenged following their child’s diagnosis [13]. These differences suggest that the stresses and impact of an acute illness such as cancer are not necessarily reflected in more chronic conditions. For a chronic illness such as NF1, couples have a longer time to adapt to the situation (often from the child’s birth). This may allow them to become more efficient in making decisions and communicating in areas pertaining to their child’s illness and acquire effective techniques over time.

Despite reports of overall relationship strength in the wake of a child’s NF1 diagnosis, findings also demonstrated a decrease in average relationship quality as compared to the year prior to the child’s diagnosis. Participants reported experiencing negative impacts on aspects of emotional and sexual intimacy after receiving their child’s diagnosis. Several factors must be considered when looking at this data. The first is to differentiate between the “strength” of the relationship versus the “quality” of the relationship, particularly the quality of the relationship prior to the diagnosis. Perhaps the “strength” of the relationship improving or being maintained is more related to working together and feeling like the relationship is a partnership, whereas the “quality” may be more related to issues such as loss of intimacy.

Next, the study tried to tease out what specific factors might play a role in the perception that the relationship changed in a negative direction. The participants identified issues that posed a stress to themselves, their partner, or their relationship. Fear of disease outcome and financial concerns were reported as the greatest stress to themselves and to their partner. In addition to changes in roles, responsibilities, emotional and physical needs, and living with uncertainty, the “what ifs” associated with disease outcome can be emotionally distressing and exhausting. Participants reported financial concerns as issues they believed to be most stressful to their partners. Having a child with a chronic condition can result in economic burden due to out-of-pocket medical expenses, lost wages, and lost career opportunities [23]. Along with the impact on the parent relationships, financial hardship has implications for both the emotional and mental health of the parent, as well as the well-being of the child. Comprehensive care must include efforts to identify and address family financial hardship [24].

If and how couples communicate with each other about these concerns can be critically important for the strength and health of the relationship. Along with specific external stresses, we explored specific problems within the relationship. Over half of the participants reported problems spending time together, staying emotionally in touch, and feeling taken for granted. When considering the relationship since the child’s diagnosis of NF1, participants also reported feeling lonelier in the relationship, a reduced sense of togetherness, and a lower degree of intimacy. Several studies with parents of children with cancer have reported the diagnosis negatively affected their level of physical intimacy and sexuality [13,25,26,27,28]. As noted above, the feeling of disconnection from one another may be a mechanism underpinning the quality of their relationship, particularly while caring for a child with chronic health care needs and can be used to explore what is happening in a parent’s relationship. It can also be used to develop support strategies to enhance communication of distress and emotional needs and strengthen the emotional connection between partners. In this regard, participants provided several suggestions for what can help strengthen the parental relationship (Table 3).

The majority of participants expressed an interest in couples counseling shortly after diagnosis and in their home communities, yet 74% were not offered this service. To increase access, counseling interventions could be integrated into the child’s treatment plan. thereby reducing barriers such as financial burden and time away from their child. The COVID-19 pandemic has demonstrated that virtual (telehealth) sessions can not only be more convenient for families, but also effective [29,30]. In addition, parents diagnosed with NF1 who are considering having more children, may benefit from reproductive genetic counseling [31]. Genetic counselors can target specific information or intervention needs.

There are currently no evidence-based couples’ interventions that target disease-specific stresses to improve parental coping skills and decrease stress resulting from chronic or acute pediatric conditions. The data from this study can be used to inform intervention strategies. One such promising intervention is the couples coping enhancement training (CCET) model. This evidence-based 3-phase cognitive–behavioral intervention that utilizes dyadic coping strategies specifically addresses couples experiencing low marital satisfaction and high marital distress [32]. Interventions utilizing dyadic coping theory and strategies have shown great importance and promise in pediatric and adult oncology populations [33].

The study is limited by the small sample size and being a single-institution study. Participants had the capacity to travel to the National Institutes of Health to participate in care for their child living with NF1 and in this study. Due to the potential impact of selection bias, the results may not fully capture the experience of having a child with NF1 on the parents’ relationship. Additionally, its cross-sectional study design and varying length of time since diagnosis makes it subject to recall bias. Both parents of only nine children participated in the study. With this small sample size of couples, we did not conduct nested analyses. Prospective, longitudinal studies and the addition of a control group can provide important insights on how the relationship is impacted at different points in time, particularly if illness severity changes and if more than one child is living with NF1. Financial issues were a reported stressor. Future studies should further explore the factors involved, possibly including lost wages from having to care for a child with a chronic illness, medication costs, or other factors. With so few parent participants being diagnosed with NF1, we were not able to explore relationship or coping differences between familial and spontaneous NF1. This would be important to explore in future studies. Other limitations include only offering the study in English, underrepresentation of racial and ethnic minorities, and overrepresentation of highly educated parents. Future studies should include perspectives from culturally and linguistically diverse parents and focus on recruiting more parent dyads. Each of these factors could influence the generalizability of the results.

There are some strengths of this study to note as well. Though men are not equally represented in the sample, they comprised over a third of the participants, a value higher than most caregiver studies. Additionally, to the best of our knowledge, this is the first study that evaluates the effects of a child’s NF1 on the parents’ relationship. Information learned from this study can contribute to the development of interventions that specifically address some of the unique stresses parents who have a child with NF1 experience in their relationship. The data may also be helpful to other parents where uncertainty of prognosis is a factor. Future research focusing on the couple’s functioning as parents and as partners, based on both parents’ reports, can best inform couple-based interventions.

## 5. Conclusions

A critical component of ongoing psychosocial care is helping parents cope with specific disease stressors. Worsening parental relationships have a significant impact on a child’s mental health and well-being. Interventions should be tailored to disease specific-stressors and in both acute and chronic populations. Prospective research will aid in better understanding how chronic conditions, such as NF1, affect caregiver’s partnerships throughout the child’s development.

## Figures and Tables

**Table 1:** Demographic Characteristics of Sample.

Demographic Characteristics	Percentage	Number
Relationship to Child		
Mother	64.0	32
Father	36.0	18
Highest Educational Level		
High School Graduate	2.0	1
Some College	6.0	3
Bachelor’s Degree	46.0	23
Master’s/Doctoral Degree	28.0	14
Other	18.0	9
Ethnicity		
Hispanic	16.0	8
Non-Hispanic	84.0	42
Race		
Asian	4.0	2
Black/African American	6.0	3
White/Caucasian	78.0	39
Other	10.0	5
Unknown/Not Reported	2.0	1
Relationship Status		
Married	84.0	42
Separated	6.0	3
Divorced	4.0	2
Living with Partner	2.0	1
Civil Union	2.0	1
Single	2.0	1
Is current partner the parent of child with NF1?		
Yes	78.0	39
No	22.0	11
Have you been diagnosed with NF1?		
Yes	12.0	6
No	88.0	44
Child’s Gender		
Male	44.0	22
Female	56.0	28
Child’s Ethnicity		
Hispanic	24.0	12
Non-Hispanic	76.0	38
Child’s Age	M = 13.84 (6–24), SD = 4.74	

**Table 2:** Stressful issues for self, partner, and relationship (*n* = 50).

Stressful Issues	For You	For Your Partner	For the Relationship
Being away from work	1 (2%)	3 (6%)	1 (2%)
Financial issues	11 (22%)	18 (36%)	17 (34%)
Lack of intimacy	1 (2%)	4 (8%)	9 (18%)
Helping child cope	4 (8%)	2 (4%)	3 (6%)
Fear of disease outcome	16 (32%)	13 (26%)	7 (14%)
Communicating with partner	4 (8%)	3 (6%)	6 (12%)

**Table 3:** Suggested Means of Strengthening the Parental Relationship.

Theme	Sample Participant Response
**Open and Respectful Communication**	*“Be compassionate about one another’s feelings.”*
	*“Give your partner permission to have a bad day.”*
	*“Be open and understanding to each other’s feelings and thoughts.”*
	*“Talk, talk, talk about how you really feel. Both sides. And listen to each other.”*
	*“Keep the lines of communication open at all times no matter what happens.”*
**Problem-Solve as a Couple, Without Blaming**	*“Don’t blame each other for the diagnosis.”*
	*“Make sure you make joint decisions.”*
	*“Do your research together.”*
	*“Both parents research and learn.”*
**Make Time for One Another Outside of the Child’s Illness**	*“Remember you started out as two. Don’t forget that.”*
	*“Focus on your child but don’t lose sight of focusing on your family as a whole. The diagnosis affects not only that child but the parents and other children as well.”*
	*“Put their relationship as a very high priority.”*
	*“Have date nights and don’t talk about what’s going on with your child’s disease/health.”*
	*“Take time for yourselves together. Remember you started out as two. Don’t forget that.”*
	*“Try to find the space and the time for their relationship.”*
**Express Gratitude**	*“I would tell them to commit to making it through the hardships, recognize each other’s contributions and thank each other for their effort to make the best of the situation.”*
	*“When we have dinner together, each of us takes a turn and say three things we are grateful for.”*
**Utilize Support from Others/Professionals**	*“Have the name of a good therapist just in case you can’t resolve your issues. Ask for help.”*
	*“Reach out to other NF families. It has really helped being able to talk with other NF moms online.”*
	*“Find support groups/individuals.”*

## Data Availability

Data from this study will be provided by the authors by request.

## Supplementary Materials

The following supporting information can be downloaded at https://www.mdpi.com/article/10.3390/children10030448/s1.
