# Supplementary material for: The Perceived Influence of Neurofibromatosis Type 1(NF1) on the Parents’ Relationship
Source: Children (Basel). 2023 Feb 25;10(3):448. doi: 10.3390/children10030448 (PMC10047031; doi:10.3390/children10030448)
Supplement: Supplementary file 1 [file children-10-00448-s001.zip › children-2207778-supplementary.pdf]

**SELF-ADMINISTERED QUESTIONNAIRE – INDIVIDUAL PARTNERS (NF1)**

*Thank you again for agreeing to participate in this study. Our goal is to learn as much as we can about what the experience of dealing with a child with NF1 is like for couples. The questionnaire begins by asking you a few basic background questions.*

1. In what year were you born? ________

2a. What is your relationship to your child with NF1?

- ☐ Biological mother
- ☐ Stepmother
- ☐ Foster/adoptive mother
- ☐ Biological father
- ☐ Stepfather
- ☐ Foster/adoptive father
- ☐ Other

2b. If Other, please describe: ______________________________________________

3a. What is the highest level of education you have finished?

- ☐ Less than high school
- ☐ Some high school
- ☐ High school graduate
- ☐ Some college/technical school/Associates degree
- ☐ Bachelors degree
- ☐ Masters/Doctoral degree
- ☐ Other

3b. If Other, please describe: ______________________________________________

4a. What is your current marital/relationship status?

- ☐ Married/Partnered
- ☐ Separated
- ☐ Divorced
- ☐ Widowed
- ☐ Living with a significant other/partner
- ☐ In a civil union
- ☐ Single
- ☐ Other

4b. If married/partnered, separated, divorced, widowed, living with a significant other/partner or in a civil union-- what year? _____________

4c. If Other, please describe: ______________________________________________

5. How old is your child? _____________

6. Is your child with NF1 a:

- ☐ Boy
- ☐ Girl

7. What is the birth month and year for your child with NF1? _______ / ________
MM YEAR

8. When was your child diagnosed with NF1? _______ / ________
MM YEAR

9a. Do you have other children?

- ☐ Yes
- ☐ No

9b. If Yes:

Age of child 1: ___________, Does this child also have NF: ☐ yes ☐ no
Age of child 2: ___________, Does this child also have NF: ☐ yes ☐ no
Age of child 3: ___________, Does this child also have NF: ☐ yes ☐ no
Age of child 4: ___________, Does this child also have NF: ☐ yes ☐ no

10. Do you or your partner/spouse have NF1?

- ☐ No
- ☐ Yes, one of us has NF1
- ☐ Yes, both of us have NF1

11a. How would you describe your ethnicity?

- ☐ Hispanic or Latino
- ☐ Not Hispanic or Latino
- ☐ Unknown or Not Reported

11b. How would you describe your race?

- ☐ American Indian or Alaska Native
- ☐ Asian
- ☐ Black or African American
- ☐ White
- ☐ Unknown or Not Reported
- ☐ Other

11c. If Other, please describe: ______________________________________________

11d. How would you describe your child's ethnicity?

- ☐ Hispanic or Latino
- ☐ Not Hispanic or Latino
- ☐ Unknown or Not Reported

11e. How would you describe your child's race?

- ☐ American Indian or Alaska Native
- ☐ Asian
- ☐ Black or African American
- ☐ White
- ☐ Unknown or Not Reported
- ☐ Other

11f. If Other, please describe: ______________________________________________

11g. How would you describe your spouse/partner's ethnicity?

- ☐ Hispanic or Latino

- ☐ Not Hispanic or Latino
- ☐ Unknown or Not Reported

11h. How would you describe your spouse/partner's race?

- ☐ American Indian or Alaska Native
- ☐ Asian
- ☐ Black or African American
- ☐ White
- ☐ Unknown or Not Reported
- ☐ Other

11i. If Other, please describe: ______________________________________________

12. Is your current spouse/partner the father/mother of your child with NF1?

- ☐ Yes
- ☐ No

13. Do your other children have the same or different parents?

- ☐ I do not have other children
- ☐ Same parents
- ☐ Different parents
- ☐ Some have the same parents, some have different parents

14. Have you been diagnosed with NF1? ☐ Yes ☐ No

15. Has your child's other parent been diagnosed NF1? ☐ Yes ☐ No ☐ Not Sure

16. How often does your family have to spend time apart for your child's treatment?

- ☐ Never
- ☐ 1 – 2 days per month
- ☐ 1 – 2 weeks per month
- ☐ 1 – 3 months per year
- ☐ More than 3 months per year

16a. Who cares for your other children if your child needs to be hospitalized?

- ☐ I do not have other children
- ☐ I care for my other children
- ☐ My spouse/partner
- ☐ Paid babysitter
- ☐ Other relative
- ☐ My child has had no need for hospitalization

16b. If other relative, is the relative paid?

- ☐ Yes
- ☐ No
- ☐ Sometimes
- ☐ Not applicable

16c. If Other, please describe: ______________________________________________

*The next part of the questionnaire was designed to better understand the impact that having a child diagnosed with NF1 can have on your relationship. In order to understand how this experience can* affect *a relationship, it is important to also understand what your relationship was like prior to your child's diagnosis.*

17. How would you describe your marriage/relationship within the year before your child's diagnosis?
- ☐ Poor
- ☐ Fair
- ☐ Good
- ☐ Very Good
- ☐ Excellent

18. How would you describe your marriage/relationship now?
- ☐ Poor
- ☐ Fair
- ☐ Good
- ☐ Very Good
- ☐ Excellent

19. Would you say: (Please check all that apply)
- ☐ I am completely satisfied with my marriage/relationship
- ☐ The future of my marriage/relationship looks promising
- ☐ I get discouraged trying to make my marriage/relationship work
- ☐ I have a good marriage/relationship with my partner
- ☐ I am concerned about the future of my marriage/relationship

20. Most persons have disagreements in their relationships. Please indicate below the approximate extent of agreement or disagreement between you and your partner for each item on the following questions.

| | Always Agree | Usually Agree | Occasionally Agree | Frequently Disagree | Usually Disagree | Always Disagree |
|---|---|---|---|---|---|---|
| Religious matters | ☐ | ☐ | ☐ | ☐ | ☐ | ☐ |
| Demonstrations of affection | ☐ | ☐ | ☐ | ☐ | ☐ | ☐ |
| Making major decisions | ☐ | ☐ | ☐ | ☐ | ☐ | ☐ |
| Sex relations | ☐ | ☐ | ☐ | ☐ | ☐ | ☐ |
| Conventionality (correct or proper behavior) | ☐ | ☐ | ☐ | ☐ | ☐ | ☐ |
| Career decisions | ☐ | ☐ | ☐ | ☐ | ☐ | ☐ |

21. How often do you and your spouse/partner do the following things:

| | All of the time | Most of the time | More often than not | Sometimes | Occasionally | Never |
|---|---|---|---|---|---|---|
| How often do you discuss or have you considered divorce, separation, or terminating your relationship? | ☐ | ☐ | ☐ | ☐ | ☐ | ☐ |
| How often do you and your partner quarrel? | ☐ | ☐ | ☐ | ☐ | ☐ | ☐ |
| Do you ever regret that you married (or lived together)? | ☐ | ☐ | ☐ | ☐ | ☐ | ☐ |
| How often do you and your spouse/partner “get on each other’s nerves”? | ☐ | ☐ | ☐ | ☐ | ☐ | ☐ |

22. Do you and your spouse/partner engage in outside interests together?

- ☐ Often
- ☐ Sometimes
- ☐ Never

23. How often would you say the following events occur between you and your spouse/partner:

| | Never | Less than once a month | 1-2 times a month | 1-2 times a week | Once a day | More often |
|---|---|---|---|---|---|---|
| Have a stimulating exchange of ideas | ☐ | ☐ | ☐ | ☐ | ☐ | ☐ |
| Work together on a project | ☐ | ☐ | ☐ | ☐ | ☐ | ☐ |
| Calmly discuss something | ☐ | ☐ | ☐ | ☐ | ☐ | ☐ |

24a. Has your child’s NF1 diagnosis impacted your marriage/relationship?
(Please choose one)

- ☐ There has been no change to our marriage/relationship
- ☐ Our marriage/relationship has gotten stronger
- ☐ Our marriage/relationship has been challenged and is not as strong
- ☐ Our marriage/relationship is in trouble, and we are considering separating
- ☐ We have separated since the diagnosis

24b. Please feel free to describe any of the above:

________________________________________________________________________________

________________________________________________________________________________

________________________________________________________________________________

25a. Conflict is natural and inevitable in marriages and other close relationships. How do you feel your marriage/relationship would be now if your child had not been diagnosed with NF1?

- ☐ The same
- ☐ Better
- ☐ Worse

25b. If you answered ‘better’, please describe how:
_______________________________________________________________________________
_______________________________________________________________________________
_______________________________________________________________________________

25c. If you answered ‘worse’, please describe how:
_______________________________________________________________________________
_______________________________________________________________________________
_______________________________________________________________________________

26a. At any point BEFORE your child’s diagnosis, did you ever seriously consider separating from your spouse/partner?

- ☐ Yes
- ☐ No

26b. If “Yes”, when did this happen? ______________________________________________

26c. Have you seriously considered separating SINCE your child’s diagnosis?

- ☐ Yes
- ☐ No

26d. If 'Yes', When did this happen? ______________________________________________

*This part of the questionnaire addresses changes that might have occurred or are occurring in your marriage/relationship based on the stress of having a child with NF1. As in the other questions, there are no ‘correct’ or right or wrong answers. We are just trying to understand your perception and experiences.*
*Please take the time to read each area outlined below and tell us if this area of your relationship is fine or needs improvement. Select an answer you think applies to your relationship right now. You will have a place to briefly tell us how you are managing this area of your life and/or about any obstacles you currently see to improving this area of your relationship.*

27. Are you and your spouse/partner emotionally connected?

- ☐ Yes, completely
- ☐ Yes, somewhat
- ☐ No, we are emotionally distant

Please check whether the items below "are a problem" or "not a problem" for you and your spouse/partner:

27a. Just simply talking to each other

- ☐ Not a problem
- ☐ Sometimes a problem
- ☐ Often a problem

27b. Staying emotionally in touch with each other

- ☐ Not a problem
- ☐ Sometimes a problem
- ☐ Often a problem

27c. Feeling taken for granted

- ☐ Not a problem
- ☐ Sometimes a problem
- ☐ Often a problem

27d. Don't feel my spouse/partner knows me very well right now

- ☐ Not a problem
- ☐ Sometimes a problem
- ☐ Often a problem

27e. Spouse/Partner is (or I am) emotionally disengaged

- ☐ Not a problem
- ☐ Sometimes a problem
- ☐ Often a problem

27f. Spending time together

- ☐ Not a problem
- ☐ Sometimes a problem
- ☐ Often a problem

27g. If things are going well in these areas (feeling emotionally connected), please tell us what you believe helps you manage this area of your lives.

____________________________________________________________________________
____________________________________________________________________________

27h. If things are not going as well as you would like, or you are feeling emotionally disengaged from your spouse/partner, please tell us what you feel is getting in the way of being able to improve this area of your relationship.

____________________________________________________________________________
____________________________________________________________________________
____________________________________________________________________________

28a. Are you and your spouse/partner handling stress effectively?

- ☐ Yes completely
- ☐ Yes somewhat
- ☐ No, we are finding it challenging to handle stress effectively

Please check whether the items below "are a problem" or "not a problem" for you and your spouse/partner:

28b. Helping each other reduce daily stresses.

- ☐ Not a problem
- ☐ Sometimes a problem
- ☐ Often a problem

28c. Talking about these stresses together.

- ☐ Not a problem
- ☐ Sometimes a problem
- ☐ Often a problem

28d. Talking together about stress in a helpful manner.

- ☐ Not a problem
- ☐ Sometimes a problem
- ☐ Often a problem

28e. My spouse/partner listening with understanding about my stresses and worries.

- ☐ Not a problem
- ☐ Sometimes a problem
- ☐ Often a problem

28f. Partner takes job or other stresses out on me.
- ☐ Not a problem
- ☐ Sometimes a problem
- ☐ Often a problem

28g. Partner takes job or other stresses out on the children or others in our life.
- ☐ Not a problem
- ☐ Sometimes a problem
- ☐ Often a problem

28h. Our child with NF1 has begun to shift preferences to want to be with or talk to one parent over the other.
- ☐ Not a problem
- ☐ Sometimes a problem
- ☐ Often a problem

28i. If things are going well in these areas (handling stress), please tell us what you believe helps you manage this area of your lives.

______________________________________________________________________________
______________________________________________________________________________
______________________________________________________________________________
______________________________________________________________________________

28j. If things are not going as well as you would like in terms of how you handle stress together, please tell us what you feel is getting in the way of being able to improve this area of your relationship.

______________________________________________________________________________
______________________________________________________________________________
______________________________________________________________________________
______________________________________________________________________________

29a. An Important event (your child's NF1 diagnosis) has occurred in your lives. I feel me and my partner/spouse are:
- ☐ Dealing with this well
- ☐ Dealing with this OK but we could be dealing with it better
- ☐ Not dealing with this well

Please check whether the items below "are a problem" or "not a problem" for you and your spouse/partner:

29b. Having very different points of view on how to handle things.
- ☐ Not a problem
- ☐ Sometimes a problem
- ☐ Often a problem

29c. Dealing with NF1 and NF1 related medical appointments has made us both irritable.
- ☐ Not a problem
- ☐ Sometimes a problem
- ☐ Often a problem

29d. Dealing with NF1 and NF1 related medical appointments has led to a lot of fighting.
- ☐ Not a problem
- ☐ Sometimes a problem
- ☐ Often a problem

29e. We are both worried about how this will all turn out.

- ☐ Not a problem
- ☐ Sometimes a problem
- ☐ Often a problem

29f. We are now taking very different positions in how we care for our child(ren).

- ☐ Not a problem
- ☐ Sometimes a problem
- ☐ Often a problem

29g. If things are going well in these areas (how you are coping with the diagnosis as a couple), please tell us what you believe helps you manage this area of your lives.

________________________________________________________________________
________________________________________________________________________
________________________________________________________________________

29h. If things are not going as well as you would like, please tell us what you feel is getting in the way of being able to improve this area of your relationship.

________________________________________________________________________
________________________________________________________________________
________________________________________________________________________

What do you believe could strengthen your marriage/relationship: (Check all that apply)

30a. My spouse/partner being more involved, specifically with:

- ☐ Medical issues
- ☐ Treatment decisions
- ☐ Household chores
- ☐ Sibling chores
- ☐ Day-to-day medical things
- ☐ Other
- ☐ Not Applicable

30b. If Other, what? ______________________________________________________

30c. My spouse/partner being less involved, specifically with:

- ☐ Medical issues
- ☐ Treatment decisions
- ☐ Household chores
- ☐ Sibling chores
- ☐ Day-to-day medical things
- ☐ Other
- ☐ Not Applicable

30d. If Other, what? ______________________________________________________

30e. If I were more involved, specifically with:

- ☐ Medical issues
- ☐ Treatment decisions
- ☐ Household chores
- ☐ Sibling chores
- ☐ Day-to-day medical things

☐ Other
☐ Not Applicable

30f. If Other, what? ______________________________________________________

30g. If I were less involved, specifically with:

☐ Medical issues
☐ Treatment decisions
☐ Household chores
☐ Sibling chores
☐ Day-to-day medical things
☐ Other
☐ Not Applicable

30h. If Other, what? ______________________________________________________

30i. My spouse/partner is comfortable with me getting out to have some time to relax/unwind

☐ Yes
☐ No

30j. My spouse/partner is comfortable with us getting out to have some time to relax/unwind

☐ Yes
☐ No

30k. My spouse/partner is comfortable getting out to have some time to relax/unwind

☐ Yes
☐ No

30l. There is nothing I believe can strengthen my marriage/relationship

☐ True
☐ False

31. *Couples find different issues that arise during their child's treatment to be more or less stressful.*
*First, check which of the following issues you would say has been stressful for you individually. (Check as many as apply)*
*Second, check which you feel has been stressful for your partner. (Check as many as apply)*
*Third, check which you feel has been stressful in your relationship. (Check as many as apply)*

| | Stressful to me | Stressful to partner | Stressful to relationship | Not applicable |
|---|---|---|---|---|
| Being away from work | ☐ | ☐ | ☐ | ☐ |
| Helping my child's siblings | ☐ | ☐ | ☐ | ☐ |
| Helping my child with medical needs | ☐ | ☐ | ☐ | ☐ |
| Financial issues | ☐ | ☐ | ☐ | ☐ |
| Lack of intimacy between my partner and I | ☐ | ☐ | ☐ | ☐ |
| Helping my child cope | ☐ | ☐ | ☐ | ☐ |
| Fear of disease outcome | ☐ | ☐ | ☐ | ☐ |
| Communicating with my partner | ☐ | ☐ | ☐ | ☐ |
| Lack of Support from my partner | ☐ | ☐ | ☐ | ☐ |

| | | | | |
|---|---|---|---|---|
| Dealing with our child's school or academic (learning) issues | ☐ | ☐ | ☐ | ☐ |
| Dealing with our child's social issues | ☐ | ☐ | ☐ | ☐ |
| Dealing with our child's Attention Deficit/Hyperactivity issues | ☐ | ☐ | ☐ | ☐ |
| Dealing with our child's psychological issues | ☐ | ☐ | ☐ | ☐ |

a) Which issue has been most stressful to you? (Select only one)

____________________________________________________________

b) Which issue has been most stressful to your partner/spouse? (Select only one)

____________________________________________________________

c) Which issue has been most stressful on your relationship? (Select only one)

____________________________________________________________

32. We want to get a sense of your marriage/relationship over the course of your child's illness. Please rate how much of the time you have felt the following items BEFORE YOUR CHILD'S NF1 DIAGNOSIS:

| | Never | Some-times | Often | Almost Always | Always |
|---|---|---|---|---|---|
| Feeling lonely in the marriage | ☐ | ☐ | ☐ | ☐ | ☐ |
| Feeling a sense of togetherness in the marriage | ☐ | ☐ | ☐ | ☐ | ☐ |
| Degree of intimacy/sexual relationship | ☐ | ☐ | ☐ | ☐ | ☐ |
| Able to make decisions together | ☐ | ☐ | ☐ | ☐ | ☐ |
| Anger | ☐ | ☐ | ☐ | ☐ | ☐ |
| Tension/stress between my partner and I | ☐ | ☐ | ☐ | ☐ | ☐ |
| Able to communicate about our ill child's needs | ☐ | ☐ | ☐ | ☐ | ☐ |

33. Now answer the same questions regarding how much of the time you have felt the following items, thinking about the impact of your child's illness over the past year (or since the diagnosis) AFTER DIAGNOSIS:

| | Never | Some-times | Often | Almost Always | Always |
|---|---|---|---|---|---|
| Feeling lonely in the marriage | ☐ | ☐ | ☐ | ☐ | ☐ |
| Feeling a sense of togetherness in the marriage | ☐ | ☐ | ☐ | ☐ | ☐ |
| Degree of intimacy/sexual relationship | ☐ | ☐ | ☐ | ☐ | ☐ |
| Able to make decisions together | ☐ | ☐ | ☐ | ☐ | ☐ |
| Anger | ☐ | ☐ | ☐ | ☐ | ☐ |
| Tension/stress between my partner and I | ☐ | ☐ | ☐ | ☐ | ☐ |
| Able to communicate about our ill child's needs | ☐ | ☐ | ☐ | ☐ | ☐ |

33h. Which issue has been most stressful to you?

________________________________________________________________
________________________________________________________________
________________________________________________________________

*The last set of questions will help us understand the time points after a child's diagnosis with NF1 that can be most challenging for couples.*

34. For the items below, rate how stressful each of the following time points have been for you personally, for your partner, and on your relationship. Next, rate the quality of your marriage/relationship at each time point.

34a. How stressful was waiting to find out what was wrong with your child for you?
- ☐ Not at all Stressful
- ☐ A little Stressful
- ☐ Stressful
- ☐ Very Stressful
- ☐ Extremely Stressful
- ☐ Not Applicable

34b. How stressful was your child's NF1 diagnosis for you?
- ☐ Not at all Stressful
- ☐ A little Stressful
- ☐ Stressful
- ☐ Very Stressful
- ☐ Extremely Stressful

34c. How stressful was making treatment decisions for you?
- ☐ Not at all Stressful
- ☐ A little Stressful
- ☐ Stressful
- ☐ Very Stressful
- ☐ Extremely Stressful
- ☐ Not Applicable

34d. How stressful was the start of treatment for you?
- ☐ Not at all Stressful
- ☐ A little Stressful
- ☐ Stressful
- ☐ Very Stressful
- ☐ Extremely Stressful
- ☐ Not Applicable

34e. How stressful were hospitalizations for you?
- ☐ Not at all Stressful
- ☐ A little Stressful
- ☐ Stressful
- ☐ Very Stressful
- ☐ Extremely Stressful
- ☐ Not Applicable

34f. How stressful were medical appointments for you?
- ☐ Not at all Stressful
- ☐ A little Stressful
- ☐ Stressful

☐ Very Stressful
☐ Extremely Stressful
☐ Not Applicable

34g. How stressful were clinical trials for you?

☐ Not at all Stressful
☐ A little Stressful
☐ Stressful
☐ Very Stressful
☐ Extremely Stressful
☐ Not Applicable

34h. How stressful was tumor progression for you?

☐ Not at all Stressful
☐ A little Stressful
☐ Stressful
☐ Very Stressful
☐ Extremely Stressful
☐ Not Applicable

34i. How stressful was being taken off treatment/clinical trial for you?

☐ Not at all Stressful
☐ A little Stressful
☐ Stressful
☐ Very Stressful
☐ Extremely Stressful
☐ Not Applicable

34j. How stressful was waiting to find out what was wrong with your child for your partner?

☐ Not at all Stressful
☐ A little Stressful
☐ Stressful
☐ Very Stressful
☐ Extremely Stressful
☐ Not Applicable

34k. How stressful was your child's NF1 diagnosis for your partner?

☐ Not at all Stressful
☐ A little Stressful
☐ Stressful
☐ Very Stressful
☐ Extremely Stressful

34l. How stressful was making treatment decisions for your partner?

☐ Not at all Stressful
☐ A little Stressful
☐ Stressful
☐ Very Stressful
☐ Extremely Stressful

☐ Not Applicable

34m. How stressful was the start of treatment for your partner?

☐ Not at all Stressful
☐ A little Stressful
☐ Stressful
☐ Very Stressful
☐ Extremely Stressful

34n. How stressful were hospitalizations for your partner?

☐ Not at all Stressful
☐ A little Stressful
☐ Stressful
☐ Very Stressful
☐ Extremely Stressful
☐ Not Applicable

34o. How stressful were medical appointments for your partner?

☐ Not at all Stressful
☐ A little Stressful
☐ Stressful
☐ Very Stressful
☐ Extremely Stressful
☐ Not Applicable

34p. How stressful were clinical trials for your partner?

☐ Not at all Stressful
☐ A little Stressful
☐ Stressful
☐ Very Stressful
☐ Extremely Stressful
☐ Not Applicable

34q. How stressful was tumor progression for your partner?

☐ Not at all Stressful
☐ A little Stressful
☐ Stressful
☐ Very Stressful
☐ Extremely Stressful
☐ Not Applicable

34r. How stressful was being taken off treatment/clinical trial for your partner?

☐ Not at all Stressful
☐ A little Stressful
☐ Stressful
☐ Very Stressful
☐ Extremely Stressful
☐ Not Applicable

34s. How stressful was waiting to find out what was wrong with your child for your relationship?

☐ Not at all Stressful

- ☐ A little Stressful
- ☐ Stressful
- ☐ Very Stressful
- ☐ Extremely Stressful

34t. How stressful was your child's NF1 diagnosis for your relationship ?

- ☐ Not at all Stressful
- ☐ A little Stressful
- ☐ Stressful
- ☐ Very Stressful
- ☐ Extremely Stressful

34u. How stressful was making treatment decisions for your relationship?

- ☐ Not at all Stressful
- ☐ A little Stressful
- ☐ Stressful
- ☐ Very Stressful
- ☐ Extremely Stressful

34v. How stressful was the start of treatment for your relationship?

- ☐ Not at all Stressful
- ☐ A little Stressful
- ☐ Stressful
- ☐ Very Stressful
- ☐ Extremely Stressful

34w. How stressful were hospitalizations for your relationship?

- ☐ Not at all Stressful
- ☐ A little Stressful
- ☐ Stressful
- ☐ Very Stressful
- ☐ Extremely Stressful
- ☐ Not Applicable

34x. How stressful was medical appointments for your relationship?

- ☐ Not at all Stressful
- ☐ A little Stressful
- ☐ Stressful
- ☐ Very Stressful
- ☐ Extremely Stressful
- ☐ Not Applicable

34y. How stressful were clinical trials for your relationship?

- ☐ Not at all Stressful
- ☐ A little Stressful
- ☐ Stressful
- ☐ Very Stressful
- ☐ Extremely Stressful
- ☐ Not Applicable

34z. How stressful was tumor progression for your relationship?

- ☐ Not at all Stressful
- ☐ A little Stressful
- ☐ Stressful
- ☐ Very Stressful
- ☐ Extremely Stressful
- ☐ Not Applicable

34aa. How stressful was being taken off treatment/clinical trial for your relationship?

- ☐ Not at all Stressful
- ☐ A little Stressful
- ☐ Stressful
- ☐ Very Stressful
- ☐ Extremely Stressful
- ☐ Not Applicable

34bb. At the time you were waiting to find out what was wrong with our child, your relationship was?

- ☐ Excellent
- ☐ Good
- ☐ Fair
- ☐ Poor

34cc. At the time of your child's NF1 diagnosis, your relationship was?

- ☐ Excellent
- ☐ Good
- ☐ Fair
- ☐ Poor

34dd. At the time of making treatment decisions for your child, your relationship was?

- ☐ Excellent
- ☐ Good
- ☐ Fair
- ☐ Poor

34ee. At the time of starting treatment for your child, your relationship was?

- ☐ Excellent
- ☐ Good
- ☐ Fair
- ☐ Poor

34ff. At the time your child's hospitalizations, your relationship was?

- ☐ Excellent
- ☐ Good
- ☐ Fair
- ☐ Poor
- ☐ Not Applicable

34gg. At the time your child's changing treatment, your relationship was?

- ☐ Excellent
- ☐ Good
- ☐ Fair

☐ Poor
☐ Not Applicable

34hh. At the time your child's tumor progression, your relationship was?

☐ Excellent
☐ Good
☐ Fair
☐ Poor
☐ Not Applicable

34ii. At the time your child finished treatment, your relationship was?

☐ Excellent
☐ Good
☐ Fair
☐ Poor
☐ Not Applicable

35. Thinking about the time points listed below, at what point did you feel closest or most emotionally connected to your spouse/partner? (Check only one)

☐ Period before the diagnosis was made
☐ When the NF1 diagnosis was made
☐ When the plexiform neurofibroma was diagnosed
☐ Enrolling on a clinical trial
☐ Making treatment decisions
☐ During hospitalizations
☐ When changing treatment
☐ Tumor progression
☐ Other

35a. If Other, please describe ___________________________________________________

36. Thinking about the time points listed below, at what point did you feel detached or least emotionally connected to your spouse/partner? (Check only one)

☐ Period before the diagnosis was made
☐ When the NF1 diagnosis was made
☐ When the plexiform neurofibroma was diagnosed
☐ Enrolling on a clinical trial
☐ Making treatment decisions
☐ During hospitalizations
☐ When changing treatment
☐ Tumor progression
☐ Other

36a. If Other, please describe:___________________________________________________

37. Thinking about the challenges listed below, please check if any of these have been a stress to your relationship with your spouse/partner.
My child’s school issues
My child’s Attention Deficit/Hyperactivity Disorder
My child’s learning disabilities

My child’s limited social skills
My child’s appearance

38a. If it was offered, would you have been interested in counseling to address ways to support/strengthen a marriage/relationship after a child is diagnosed with NF1?

- ☐ Yes
- ☐ No
- ☐ Not sure

38b. If yes or not sure, when would have been the best time to be offered this type of support?

- ☐ Soon after my child’s diagnosis
- ☐ In the first year after my child’s diagnosis
- ☐ At any point after my child’s diagnosis when we might benefit
- ☐ Only if we ask for this service
- ☐ It should never be offered
- ☐ Other

38c. If Other, please describe:___________________________________________________

39. What topics relating to your child’s diagnosis and your marriage/relationship are most important to address in a counseling/supportive intervention?

______________________________________________________________________________
______________________________________________________________________________
______________________________________________________________________________

40. If offered, where would you be more likely to use this service:

- ☐ At the hospital where your child is being treated
- ☐ In your home community (mental health clinic or private practice)
- ☐ At a church/place of worship
- ☐ Other

40a. If Other, please describe:__________________________________________________

41a. What might keep you from participating in a counseling/supportive marital intervention? (Check all that apply)

- ☐ Limited time
- ☐ Costs too much
- ☐ My partner would never do it
- ☐ Difficult for both my partner and I to be at the hospital at the same time
- ☐ I worry talking about our relationship issues will be too difficult to deal with while my child is receiving treatment
- ☐ I’m not interested
- ☐ I don’t feel we need it
- ☐ Other

41b. If Other, please describe:___________________________________________________

42a. Have you or your spouse/partner ever been offered marital support/counseling since your child was diagnosed with NF1?

- ☐ Yes
- ☐ No

42b. If 'Yes', by whom: _________________________________________________________

43a. Have you or your spouse/partner ever used marital support/counseling prior to your child being diagnosed with NF1?

☐ Yes
☐ No

43b. If 'Yes', this was to help with: _______________________________________________

44a. Would you be interested in counseling/support that was not face-to-face, like telephone or online video-conferencing support (e.g., like SkypeTM)

☐ Yes
☐ No

44b. If 'No', why not: __________________________________________________________________

45. How worried are you about your child's illness?

☐ Not at all worried
☐ A little worried
☐ Moderately worried
☐ Very worried
☐ Extremely worried

45a. What do you think about your child's prognosis? (Please check one)

☐ I am not sure
☐ I think my child will survive with none or very limited complications related to his/her NF1
☐ I think my child will survive, but will have complications
☐ I think that my child will not survive due to complications from his/her NF1
☐ Other

45b. If 'Other', please specify: ___________________________________________________

46a. If your child's NF1 did progress and there was little chance of cure, which statement best describes how you might respond:

| | |
|---|---|
| ☐ | I would be interested in available treatment if it could possibly extend my child's life |
| ☐ | I would be interested in available treatments that could possibly extend my child's life as long as I felt that it wouldn't make my child's quality of life worse |
| ☐ | I would want to focus on providing comfort care primarily |
| ☐ | I think my child's disease might come back in the next few years |
| ☐ | Other |

46b. If 'Other', please specify: ___________________________________________________

46c. Do you think you and your partner would agree?

☐ Yes
☐ No
☐ Not sure

47. Is there an event or time that stands out in your mind that helped bring you closer together? (e.g., something that your spouse/partner did/said; something that your child did/said; something that someone else did/said: a realization on your own). If so, please tell us what it was and when during your child's treatment this occurred.

_____________________________________________________________________________
_____________________________________________________________________________

48. Is there an event or time that stands out in your mind when you felt most distant from your partner/spouse? (e.g., something that your spouse/partner did/said; something that your child did/said; something that someone else did/said: a realization on your own). If so, please tell us what it was and when during your child's treatment this occurred.

______________________________________________________________________________
______________________________________________________________________________
______________________________________________________________________________
______________________________________________________________________________

49. Is there an event that stands out in your mind when you felt most angry at your spouse?

______________________________________________________________________________
______________________________________________________________________________
______________________________________________________________________________
______________________________________________________________________________

50. What recommendations would you make to a couple whose child was recently diagnosed in terms of trying to keep their relationship strong?

______________________________________________________________________________
______________________________________________________________________________
______________________________________________________________________________
______________________________________________________________________________
______________________________________________________________________________

Were there topics you thought would be covered, but were not? If so, please feel free to let us know. Any other feedback is welcome as well.

______________________________________________________________________________
______________________________________________________________________________
